# Automating content analysis of scientific abstracts using ChatGPT: A methodological protocol and use case

**DOI:** 10.1016/j.mex.2025.103431

**Published:** 2025-06-13

**Authors:** Adrián Domínguez-Diaz, Manuel Goyanes, Luis de-Marcos

**Affiliations:** a Computer Science, Universidad de Alcalá, Madrid, Spain; b Communication, Universidad Carlos III de Madrid, Madrid, Spain

**Keywords:** ChatGPT, Content analysis, Protocol, Scientific abstracts, Research approach, Data collection method, Large language model, Content Analysis using ChatGPT

## Abstract

This paper presents a protocol for using ChatGPT to perform content analysis. The protocol involves converting a codebook, outlining categories, descriptions, coding rules, and possible values, into a structured prompt that guides ChatGPT's analysis. The protocol was validated through analysis of 980 research articles to identify research approaches and data collection methods. ChatGPT achieved high performance in identifying data collection methods, but faced challenges with poorly defined or underrepresented categories, particularly in mixed methods research. Overall, while it scored well for quantitative (0.96) and qualitative (0.82) studies, it struggled with mixed methods (0.60), highlighting the need for clear methodological definitions.

• The protocol enhances coding efficiency and demonstrates the feasibility of using AI for content analysis, potentially streamlining the coding process in research.
• Challenges arose in categories that were not clearly defined (big data), underrepresented (ethnography), or hierarchically related (Interview & Discourse/Textual analysis).
• Interrater metrics indicated a substantial level of agreement, reinforcing the potential of ChatGPT in content analysis while emphasizing the importance of clear methodological definitions.

## Graphical abstract

**Figure:**
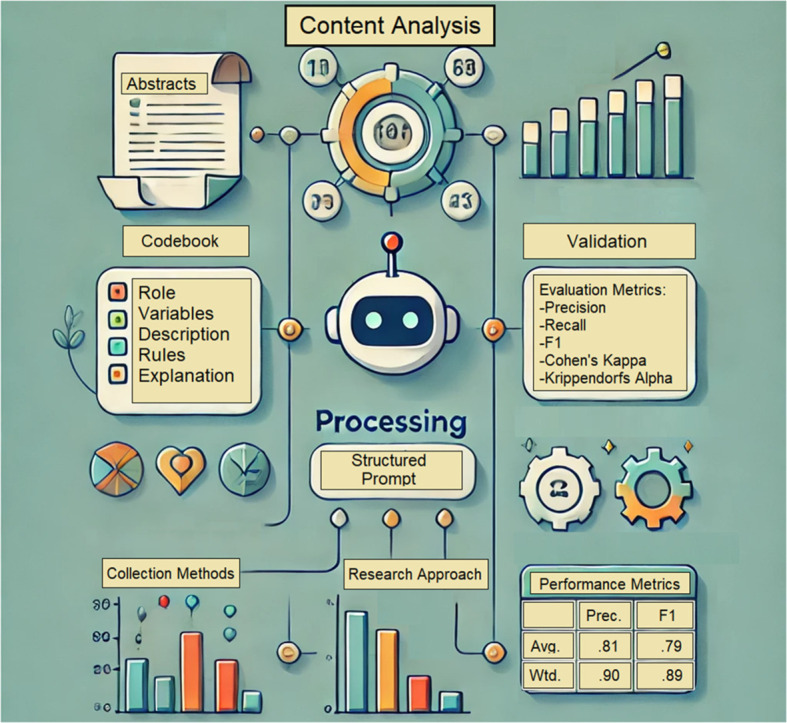

Specifications table

**Table:** 

Subject area:	Computer Science
More specific subject area:	Automatic coding
Name of your method:	Content Analysis using ChatGPT
Name and reference of original method:	None
Resource availability:	None

## Background

For the generation of scientific knowledge, scholars have access to a wide range of methodologies, spanning from qualitative to quantitative approaches. Within these scientific frameworks, particularly in the social sciences, scholars rely on various methodological processes to collect evidence, infer results, and, through hypothesis testing, contribute to the advancement of knowledge. Different scientific fields often rely on distinctive methodologies, which, due to their widespread use and accessibility, not only define the field itself but are also fundamental for understanding the phenomena and objects of study within that discipline.

In the social sciences, and particularly in communication studies, one of the most important methodologies for advancing knowledge and theories about communicative processes is content analysis [6,10,16]. According to numerous prior studies [4,11], content analysis is one of the most widely used methodologies in this field because it allows for the systematic and objective study of text and messages in different types of formats, including print, television, social media, and academic publications [7]. Content analysis stands out as a key research method in communication because it is easy to use, thanks to decades of refinement, accessible to researchers with limited resources, and highly effective for systematically, objectively, and quantitatively analyzing media messages [12,13]. It best represents the nature, origins, and evolution of communication research.

Despite its significant influence in communication studies, content analysis has broadened its scope and is now utilized across a diverse array of disciplines, including but not limited to psychology, political science, education, and related fields. Content analysis is increasingly being applied in other “hard” sciences, although it is often employed without adhering to the rigorous methodological frameworks established in communication research. For instance, in fields such as computer science—particularly in domains like information retrieval—manual coding is commonly performed. However, its use is rarely acknowledged or systematically documented. These efforts rarely adopt a formalized approach that includes the development of a clear codebook, the validation of categories, or inter-coder reliability testing, which are central elements of content analysis in communication. This can lead to subjective or inconsistent interpretations, as coding decisions often depend on the individual judgment of raters without the support of an explicit protocol.

Moreover, the lack of procedures such as the calculation of reliability indices (e.g., Krippendorff’s Alpha or Cohen’s Kappa) increases the risk of producing results that are less replicable and more prone to human error. Therefore, while content analysis has cross-disciplinary applications, the methodological standards of communication studies provide a more robust framework for minimizing biases and ensuring the quality of results. This underscores the need to extend these methodological best practices to other disciplines that use content analysis in a less structured manner.

The traditional process of content analysis was typically conducted through human coding [1,2,8,9,15,18]. In this process, two or more independent coders analyze the content to establish judgments based on pre-training with a coding book provided for categorizing the variables. Following the independent coding, a reliability test is performed to assess whether the codifications made by the coders achieve acceptable levels of reliability. This traditional approach to human coding is often regarded as time-consuming and resource-intensive, requiring significant human involvement, including multiple coders and considerable time [14]. However, with the advent of artificial intelligence platforms, the processes associated to the content analysis can be streamlined and simplified, potentially achieving levels of reliability comparable to or even surpassing those of human coders.

However, despite the significant potential of artificial intelligence tools to facilitate and enhance the process of content analysis, there is a notable lack of empirical and theoretical studies that have systematically designed, tested, and validated a research protocol for using these platforms in this context. ChatGPT, as a natural language processing model, represents a key innovation for the future of automated text coding, as it has the potential to simplify and expedite the coding of large text datasets while requiring fewer time and economic resources. Moreover, ChatGPT is already, and could continue to be, a technology for automating content analysis, streamlining researchers’ workflows, and increasing their productivity. With this in mind, the objective of this study is to propose a research protocol that outlines the steps and best practices for conducting content analysis with the assistance of ChatGPT. Additionally, we aim to evaluate the outputs generated by ChatGPT in comparison with those produced through traditional human coding. The ultimate goal is to provide a protocol that future scholars interested in this methodology can adopt and refine for text analysis.

To achieve this, the proposed protocol involves translating a codebook—containing categories, descriptions, coding rules, and possible values—into a structured prompt that guides ChatGPT’s analysis. We demonstrate the protocol by applying it to a dataset of 980 communication research articles, classifying the research approach (quantitative, qualitative, or mixed) and identifying the primary data collection methods used. To guide this investigation, we pose the following research questions:

RQ1: How effectively can ChatGPT be utilized for content analysis of scientific articles, particularly in identifying the primary research method employed and classifying the research approach?

RQ2: What is the level of agreement between ChatGPT's coding of research approach (quantitative, qualitative, or mixed) and data collection methods, compared to the coding performed by human content analysts?

By addressing these questions, we aim to contribute to the ongoing dialogue about the role of AI in academic research and to provide empirical evidence regarding the potential of ChatGPT as a tool for content analysis of scientific production. This study aligns with recent bibliometric analyses of ChatGPT research in specific fields, such as medicine [3], nursing education [17] and media [5], which highlight the growing interest in understanding and leveraging AI technologies across academic disciplines.

## Method details

### Protocol

The protocol defines how to convert a codebook that specifies the variables to be analyzed and coded into a prompt that instructs the language model to perform content analysis. The codebook includes a list of variables to be analyzed, and each variable may have a description, a list of possible coding values, and coding rules. This protocol generates a prompt for ChatGPT to analyze and interpret text, assigning values to each variable and, optionally, indicating the parts of the text used to infer its decisions. For this protocol, we use computing terminology—such as variables, values, and rules—instead of content analysis terminology, which can be inconsistent. Variables are sometimes referred to as categories, themes, or codes, while values may also be called codes or labels. Since categorical variables are commonly coded within a set of categories, their coding values represent a coding category. In this paper we use *category* to refer to coding categories of categorical variables. Table 1 presents the coding book used to extract the research method and research approach from the abstracts of scientific papers, defining two variables.

**Table 1:** Codebook for classification of research approach and research method.

#	Item	Content
1	Variable name	research_approach
	Description	indicates whether the research is Quantitative, Qualitative or Mixed
	Rules	If the study has just one research method, it should be classified as Quantitative or Qualitative. If the study has more than one research method, it should be classified as Mixed
	Explanation	No
2	Variable name	research_method
	Description	indicates the type of the main research method used to collect data
	Values	Methodological advances, Systematic literature review, Meta-analysis, Big data, Experimental, Ethnography, Observational analysis, Case study, Discourse and textual analysis, Focus group, Interview, Survey, Content analysis, Network analysis
	Rules	The values have to be ordered by descending priority, in case more than one research method is detected
	Explanation	Yes

*Note*: Each item represents a variable with its name (Variable), description, values, and rules. Values and rules are optional. This research uses computing terminology. In content analysis terminology a Variable can also be called Theme, Code or Category. Values can also be called labels.

The protocol (Table 2) begins by instructing ChatGPT on its role and the task to be performed. Under ‘{document_description}’, the type of document is identified. For this research, we used ‘abstract’. Then, for each variable defined in the codebook, the protocol directs the LLM to code it using the description, values, and rules, if provided. If the description specifies the possible values, it is not necessary to present them again. Rules can further clarify coding instructions, as illustrated in the example provided in Table 1. When the codebook requires an explanation for a given variable, the protocol instructs ChatGPT to create a new variable named ‘{variable_name_description}’ and return the fragments of the original text used to make the coding decision. The protocol concludes by indicating that all variables are defined and providing the text to be analyzed.

**Table 2:** Protocol for content analysis using ChatGPT.

#	Description	Instruction
1	Role	You will have the role of a researcher that performs content analysis tasks.
2	Task	I need you to code a series of variables based on {document_description}. Next, I will describe you the variables.
3	For each variable	Variable “{variable_name}” which {variable_description}.
	If values	Answer with one of the following values: {values}
	If rules	{rules}
	If explanation	Variable "{variable_name}_explanation” where you return an array with the text fragments used to determine the value of "{variable_name}". Include up to 10 words per text fragment.
4	Ending	All the variables are defined. This is the content to analyze (between triple quotes): """{text_content}""" Return the response in JSON format. Do not include the content itself in the response.

*Note*: This protocol translates a codebook described in the form presented in Table 1 to instructions for ChatGPT LLM. Values and rules for each variable are optional.

This protocol can be applied through both ChatGPT's chat mode and the OpenAI API. In chat mode, ChatGPT responds to each message step-by-step, confirming the process as defined in the protocol. The process is entirely guided, ensuring that ChatGPT receives all necessary information and knows what to return at each stage. To apply the protocol programmatically, the *client.chat.completions.create* function is used with the ChatGPT 3.5-turbo model. In the initial message to the LLM, the role is defined under system, while subsequent messages are separated by line breaks under user. Additionally, the model’s temperature is set to 0, as creativity is not required for this task. To facilitate further programmatic processing of the output, we suggest requesting the response in JSON format. Annex 1 presents the Python source code implementing the protocol using the ChatGPT API.

### Test case

As an illustration of the content analysis procedure using ChatGPT, we present a use case where it is applied to analyze abstracts of scientific articles. We collected a dataset of 980 scientific articles in the field of communication. The dataset was coded by two human content analysts based on the article abstracts using the codebook previously described (Table 1). Content analysts coded the complete dataset independently and met later only to solve discrepancies. Data collection methods were classified under 13 categories (e.g., experimental, interview, content analysis) identifying only the primary data collection method used in the study. Categories were ordered hierarchically, instructing coders and the model to prioritize items in higher positions when multiple responses were possible. Categories on ‘text analysis’ and ‘discourse analysis’ were grouped under a single category since coders did it given their methodological similarities for the sample under analysis. The research approach was coded as quantitative, qualitative, or mixed. The codebook instructs analysts to code the approach as mixed when more than one research method is used, which may differ from the traditional understanding of mixed methods (combination of quantitative and qualitative methods) [19]. For systematic literature reviews, the approach was coded as empty and the data collection method as ‘systematic literature review’. Descriptive data of the two coding variables of the dataset are presented in Table 3.

**Table 3:** Descriptive data of the test case of 980 research papers on communication.

Research approach	Data collection method	N
Qualitative	Interview	92
(n = 173)	Discourse analysis / textual analysis	54
	Focus group	17
	Ethnography	8
	Case study	2
Quantitative	Survey	306
(n = 686)	Experimental	252
	Content analysis	78
	Big data	24
	Meta-analysis	17
	Observational analysis	5
	Methodological advances	3
	Network analysis	1
Mixed Methods (n=107)	–	107
(Empty) (n = 14)	Systematic literature review	14

*Note*: Papers were classified under research approach and the main data collection method based on their abstracts.

### Metrics

The following performance metrics were computed for each coding category of each variable: precision, recall, and F1-score. Precision is the ratio of true positive results to the total number of positive results predicted by the model. It measures the accuracy of the positive predictions. Recall is the ratio of true positive results to the total number of actual positives. It measures the model’s ability to identify all relevant instances. F1-score is the harmonic mean of precision and recall, providing a single metric that balances both concerns. Agreement of human coders was taken as the ground truth to compute precision, recall and F1-score. Overall versions of these metrics (averaged and weighted) were also computed for the complete dataset. Additionally, global reliability metrics, including Cohen’s Kappa and Krippendorff’s Alpha, were also computed. Cohen’s Kappa (weighted) is a a statistical measure that assesses inter-rater agreement for categorical items, correcting for agreement occurring by chance. Krippendorff’s Alpha is a measure of the agreement among raters, applicable to any number of raters and different levels of measurement, including nominal, ordinal, interval, and ratio scales. For research approach, records labeled as empty were excluded from the computation of metrics. Additionally, categories containing five or fewer records in the dataset were also excluded to compute overall metrics to avoid introducing biases by underrepresented categories.

Most frequent discrepancies between human coders and ChatGPT in data collection methods were identified by counting the number of false positives and false negatives. For each category (cat) in which a false positive or negative was made involving an erroneous category (cat_err), the error frequency was calculated using the following formulas:

$$\%FP_{cat\_err}\;=\;FP_{cat\_err}\;/\;\left(TP_{cat}+\;FP_{cat}\right)$$

$$\%FN_{cat\_err}\;=\;FN_{cat\_err}\;/\;\left(TP_{cat}+\;FN_{cat}\right)$$

where $FP_{cat\_err}$ is the number of records from cat_err incorrectly coded cat, $FN_{cat\_err}$ is the number of records from category cat incorrectly coded as cat_err, and $TP_{cat}$, $FP_{cat}$ and $FN_{cat}$ are the total number of true positives, true negatives and false negatives of category cat.

## Method validation

### Quantitative metrics

Table 4 presents the metrics for ChatGPT's ability to identify the primary data collection methods across various categories of scientific abstracts. Categories such as ‘Meta-analysis’ and ‘Focus group’ exhibit exceptionally high performance, achieving F1-scores of 0.97 and 0.94, respectively, indicating reliable categorization. However, methods like ‘Big data’ have significantly lower performance, with an F1-score of only 0.26 due to its low recall (0.17). Overall, the average F1-score across all categories is 0.79, and the weighted average is 0.84, demonstrating a moderate-to-high level of effectiveness in identifying data collection methods. The reliability of ChatGPT in categorizing the data collection methods was assessed using two inter-rater reliability metrics: Cohen's Kappa and Krippendorff's Alpha. The Cohen's Kappa score of 0.78 indicates a substantial level of agreement between ChatGPT and human coders, suggesting that its classifications are consistent and dependable. Similarly, the Krippendorff's Alpha score of 0.76 further validates the model's reliability, confirming its robustness in maintaining consistent performance across diverse data collection categories.

**Table 4:** Performance metrics for the main data collection method.

Category	Precision	Recall	F1-Score
Meta-analysis	0.94	1.00	0.97
Focus group	1.00	0.88	0.94
Experimental	0.93	0.85	0.89
Systematic literature review	1.00	0.79	0.88
Interview	0.92	0.79	0.85
Survey	0.88	0.82	0.85
Content analysis	0.71	0.87	0.78
Ethnography	0.70	0.88	0.78
Discourse / textual analysis	0.76	0.73	0.75
Big data	0.57	0.17	0.26
Average	0.84	0.78	0.79
Weighted	0.87	0.81	0.84

*Note*: Categories containing five or fewer records in the dataset were excluded

Table 5 reports performance metrics for identifying research approaches (quantitative, qualitative, mixed). ChatGPT excels in recognizing quantitative approaches, achieving an F1-score of 0.96 with high precision and recall. For qualitative research, while precision is lower (0.74), recall is strong (0.91), resulting in an F1-score of 0.82. The performance for mixed approaches lags behind, with an F1-score of 0.60, mainly due to lower recall (0.51). The weighted F1-score of 0.89 across all categories underscores its reliable performance, though improvement is needed for mixed approaches. The reliability of the research approach was similarly evaluated using Cohen's Kappa and Krippendorff's Alpha. The Cohen's Kappa score of 0.76 demonstrates substantial agreement between ChatGPT and human analysts in classifying research approaches as quantitative, qualitative, or mixed. This is closely mirrored by the Krippendorff's Alpha score of 0.77, reinforcing the model’s reliability in this domain. These metrics collectively highlight ChatGPT's potential as a reliable tool for automating content analysis tasks, though slight improvements could enhance alignment with human coders. Table 4, Table 5 in combination illustrate ChatGPT's robust performance in content analysis, with weighted F1-scores above 0.80 for both research approaches and data collection methods.

**Table 5:** Performance metrics for research approach.

Category	Precision	Recall	F1-Score
Quantitative	0.96	0.95	0.96
Qualitative	0.74	0.91	0.82
Mixed	0.71	0.51	0.60
Average	0.81	0.79	0.79
Weighted	0.90	0.89	0.89

### Error analysis

Table 6 presents common false-positive errors, categorized by the primary data collection method. Big data is often misclassified as Content analysis (28.6% of errors). ‘Content analysis’ itself suffers from misclassification with ‘Survey’ (9.5%) and ‘Discourse/Textual Analysis’ (8.4%) as frequent sources of confusion. Other notable errors include ‘Discourse/Textual Analysis’ being misclassified as ‘Interview’ (13%) and ‘Ethnography’ being confused with ‘Interview’ (20%). These patterns reveal areas where the model struggles with overlapping methodological features.

**Table 6:** Most frequent errors. False positives (>1%) for data collection methods.

Category	Error#1	Error#2	Error#3
Big data	Content analysis (28.6%)	-	-
Content analysis	Survey 9.5%)	Discourse / textual analysis (8.4%)	Big data (5.3%)
Discourse / textual analysis	Interview (13%)	Survey (3.7%)	Ethnography (1.9%)
Ethnography	Interview (20.0%)	Discourse / textual analysis (10.0%)	-
Experimental	Survey (4.8%)	Big data (1.3%)	-
Focus group	-	-	-
Interview	Experimental (3.8%)	Survey (2.5%)	Focus group (1.3%)
Meta-analysis	Survey (5.6%)	-	-
Survey	Experimental (8.8%)	Interview (1.8%)	-
Systematic literature review	-	-	-

*Note*: Columns Error#1, Error#2 and Error#3 represent the most common errors between categories measured as the percentage of false positives when it is larger than 1%. Empty cells mean no errors between the category and others.

Table 7 highlights the most common false negatives, where specific methods were not identified correctly. For instance, ‘Big data’ is frequently missed, being instead classified as ‘Network Analysis’ (29.2%) or ‘Content Analysis’ (20.8%). ‘Content analysis’ is sometimes incorrectly excluded, with ‘Observational Analysis’ and ‘Survey’ as alternative categorizations (2.6% each). Misclassification trends suggest difficulty distinguishing between methodologies that share thematic or procedural similarities, such as ‘Discourse/Textual Analysis’ being confused with ‘Content Analysis’ (14.3%) and ‘Ethnography’ with ‘Discourse/Textual Analysis’ (12.5%). Errors documented in Table 6, Table 7 reflect challenges in differentiating nuanced methodologies, particularly between conceptually adjacent categories like qualitative approaches and certain data collection techniques.

**Table 7:** Most frequent errors. False negatives (>1%) for data collection methods.

Category	Error#1	Error#2	Error#3
Big data	Network analysis (29.2%)	Content analysis (20.8%)	Observational analysis (16.7%)
Content analysis	Observational analysis (2.6%)	Survey (2.6%)	Big data (2.6%)
Discourse and textual analysis	Content analysis (14.3%)	Observational analysis (3.6%)	Narrative thematic analysis (1,8%)
Ethnography	Discourse / textual analysis (12.5%)	-	-
Experimental	Survey (9.9%)	Observational analysis (2.8%)	Interview (1.2%)
Focus group	Discourse / textual analysis (5.9%)	Interview (5.9%)	-
Interview	Discourse / textual analysis (7.6%)	Survey (5.4%)	Ethnography (2.2%)
Meta-analysis	-	-	-
Survey	Observational analysis (5.2%)	Experimental (3.6%)	Content analysis (2.9%)
Systematic literature review	Content analysis (21.4%)	-	-

*Note*: Columns Error#1, Error#2 and Error#3 represent the most common errors between categories measured as the percentage of false negatives when it is larger than 1%. Empty cells mean no errors between the category and others.

## Limitations

This work expands on the themes and methodologies presented in the previous literature. Similar to the findings of Chew et al. [5] and Bijker et al. [3], this work focuses on automating content analysis using ChatGPT, reinforcing the growing interest in utilizing AI for this purpose. Our results specifically target scientific abstracts, providing a practical application in a domain highly relevant to academic research. This work presents a detailed research protocol for implementing ChatGPT in content analysis, a feature absent in the other sources. It outlines clear steps for converting a codebook into a structured prompt, guiding ChatGPT's analysis. Additionally, our findings rigorously evaluate the protocol's effectiveness using various performance metrics (precision, recall, F1-score, Cohen's Kappa, Krippendorff's Alpha), providing quantifiable evidence of ChatGPT's capabilities and limitations. This contributes to a more systematic and transparent approach to AI-assisted content analysis, addressing the need for standardized procedures and evaluation methods pointed out by Yalcinkaya and Yucel [17]. This paper concentrates on analyzing two key elements of research articles: research approach (quantitative, qualitative, or mixed) and primary data collection methods. This focus complements the broader exploration of content analysis applications in nursing education by Yalcinkaya and Yucel [17] and in medicine by Bijker et al. [3]. By targeting these specific elements, this work provides insights into ChatGPT's capacity to handle nuanced methodological distinctions within academic research, an area less explored in the other sources.

### Misclassifications

By combining quantitative metrics (Table 4, Table 5) with error analysis (Table 6, Table 7), the results provide a comprehensive view of ChatGPT's strengths and limitations in automating the content analysis of scientific abstracts. The results reveal significant challenges in accurately classifying the ‘Big Data’ category, which is characterized by very low sensitivity. ChatGPT frequently misclassifies papers labeled as ‘Big Data’ by human coders, often assigning them to other categories. This difficulty is understandable, as ‘Big Data’ does not represent a data collection method but rather refers to the volume of data used in a study. Each study categorized as ‘Big Data’ inherently involves another underlying data collection method. Consequently, it is expected that the model would struggle to assign ‘Big Data’ accurately in many instances.

Two other categories with comparatively low F1 scores are ‘Content Analysis’ and ‘Discourse/Textual Analysis.’ Analysis of frequent errors shows that ChatGPT often misassigns ‘Content Analysis’ to documents originally classified as ‘Discourse/Textual Analysis’ (9.4%). Conversely, the ‘Discourse/Textual Analysis’ category shows a high rate of false negatives with ‘Content Analysis’ (14.3%), significantly reducing both precision and sensitivity. This suggests that ChatGPT struggles to distinguish between these methods, particularly when the studies focus on analyzing textual documents. Additionally, the ‘Discourse/Textual Analysis’ category includes two somehow distinct methods -‘Discourse Analysis’ and ‘Textual Analysis’- which further complicates classification. A manual review of ChatGPT's responses compared to human coding reveals that most errors involve confusion with ‘Textual Analysis’ rather than ‘Discourse Analysis,’ indicating a nuanced area of misclassification.

For the ‘Ethnography’ category, precision and sensitivity metrics are not representative due to class imbalance, as this category contains only eight records in the dataset. With such limited representation, even a small number of errors accounts for a significant percentage. This suggests that the ‘Ethnography’ category may not be sufficiently represented for inclusion in overall classification metrics. Nonetheless, observed errors often involve ambiguous cases where the ethnographic method overlaps with others, such as ‘Interview’ or ‘Discourse Analysis.’ Finally, a review of frequent errors in other categories highlights difficulties in cases where there is a hierarchical relationship between methods. For example, misclassifications occur between ‘Discourse/Textual Analysis’ and ‘Interview,’ Content Analysis’ and ‘Survey,’ ‘Experimental’ and ‘Survey,’ and ‘Systematic Literature Review’ and ‘Content Analysis.’ In these cases, the methods in question often build upon or rely on data collected through other techniques. While this issue was partially mitigated by refining the prompt to instruct the model to prioritize methods based on a predefined hierarchy, it still notably affects classification results.

### Explicit Instructions and prioritization

The refinement of prompts, including providing explicit instructions and prioritization of methods, influences ChatGPT's performance. Evidence from the error analysis shows that ChatGPT struggles particularly in hierarchical and overlapping categories, such as those involving ‘Content Analysis’ and ‘Survey’ or ‘Discourse/Textual Analysis’ and ‘Interview.’ By incorporating prompts that prioritize assigning methods according to a predefined hierarchy, the model's misclassification rates in these areas were partially mitigated.

This suggests that tailored prompts can help the model navigate ambiguous cases by focusing on explicit relationships between methods. While the improvement is not sufficient to eliminate all errors, it indicates that explicit instructions can enhance accuracy and potentially reduce false positives and negatives in categories with conceptual overlap or hierarchical dependency.

### Influence of hierarchical relationships on coding

The results directly highlight how ChatGPT's understanding of hierarchical relationships between methods influences its coding decisions. For instance, in cases where methods are inherently interdependent (e.g., ‘Systematic Literature Review’ relying on ‘Content Analysis’), ChatGPT often errs by misclassifying the broader method as its underlying process. Similarly, the frequent confusion between ‘Discourse/Textual Analysis’ and ‘Content Analysis’ suggests that ChatGPT struggles to disentangle overlapping methodological characteristics, especially when studies involve textual data.

These observations suggest that ChatGPT's internal logic does not fully align with the hierarchical relationships or nuanced distinctions between certain methods. However, this limitation was somewhat alleviated when prompts explicitly instructed the model to respect prioritization rules or clarified the boundaries between categories. Thus, while ChatGPT's inherent understanding of these relationships is limited, carefully designed prompts can guide its decision-making and improve alignment with human coders’ interpretations. This highlights the importance of leveraging explicit instructions to address inherent challenges in dealing with hierarchical and overlapping research methods.

## Implications

Our protocol highlight the potential use of ChatGPT as a tool to support or partially automate content analysis in research. ChatGPT demonstrated moderate-to-high reliability in identifying research approaches (e.g., quantitative, qualitative, or mixed) and certain data collection methods, particularly those with distinct and well-defined characteristics like ‘Meta-analysis’ or ‘Focus Groups.’ With weighted F1-scores exceeding 0.8 for many categories, ChatGPT shows promise as a supportive tool for large-scale content analysis, particularly when human resources are limited or efficiency is a priority. Despite its limitations, ChatGPT’s performance in automated coding tasks offers scalability that is difficult to achieve with human coders alone. For research projects involving the analysis of large datasets, ChatGPT could be employed as a first-pass tool to pre-classify abstracts or articles, allowing human analysts to focus their efforts on reviewing and correcting more challenging cases. This hybrid approach could save considerable time and resources.

Findings are relevant to both researchers and practitioners in the field of content analysis. For researchers, the demonstrated protocol provides a systematic approach to leveraging AI tools like ChatGPT for content analysis, potentially enhancing the efficiency and accuracy of coding large datasets. The insights gained from the case study highlight the importance of clear definitions and distinctions among research methods, which can guide future research design and methodology training. Furthermore, the varying performance across different categories underscores the need for continued refinement of prompts and coding frameworks when employing AI for nuanced tasks, particularly in mixed methods research. For practitioners, the findings suggest that AI-driven content analysis can serve as a valuable tool for media analysts and journalists seeking to understand trends in research outputs and methodologies. By adopting this protocol, practitioners can expedite their analysis processes, enabling them to focus on interpretation and application of findings rather than manual coding. Additionally, the study's insights into the limitations of AI in capturing certain research approaches may inform training programs for practitioners, emphasizing the necessity of critical engagement with AI outputs.

Overall, this work contributes to the methodological discourse surrounding AI in research and also opens avenues for future studies to explore the integration of AI tools in various research domains, fostering a more nuanced understanding of their capabilities and limitations. Our protocol demonstrate the potential of ChatGPT for content analysis but also highlight challenges and limitations. Careful consideration must be given to defining clear and distinct categories, addressing hierarchical relationships between methods, ensuring balanced datasets for evaluation, and refining prompts to improve accuracy in challenging areas.

## Ethics statements

None.

## CRediT authorship contribution statement

**Adrián Domínguez-Diaz:** Conceptualization, Methodology, Software, Validation, Formal analysis, Investigation, Resources, Data curation, Writing – original draft, Writing – review & editing. **Manuel Goyanes:** Conceptualization, Methodology, Validation, Formal analysis, Investigation, Writing – original draft, Writing – review & editing, Supervision. **Luis de-Marcos:** Conceptualization, Methodology, Formal analysis, Resources, Data curation, Writing – original draft, Writing – review & editing, Visualization, Supervision.

## Declaration of competing interest

The authors declare that they have no known competing financial interests or personal relationships that could have appeared to influence the work reported in this paper.

## Data Availability

Data will be made available on request.

## Appendix Supplementary materials

Image, application 1
