# Supplementary material for: Automating content analysis of scientific abstracts using ChatGPT: A methodological protocol and use case
Source: MethodsX. 2025 Jun 13;15:103431. doi: 10.1016/j.mex.2025.103431 (PMC12221365; doi:10.1016/j.mex.2025.103431)
Supplement: Supplementary file 1 [file mmc1.docx]

| *Python source code to implement the protocol using the ChatGPT API* |
| --- |
| from openai import OpenAI  import json  import os  client = OpenAI(api_key="API KEY HERE")  # For each ".txt" file in the chatgpt_text folder  input_file = 'data/abstracts/abstracts_used.json'  output_file = 'data/abstracts/papers_gpt35_protocol.json'  # Read input_file as json  with open(input_file, 'r') as f:  papers = json.load(f)  try:  with open(output_file, 'r') as f:  output = json.load(f)  except:  output = {}  # Create and output json with a dictionary for each paper  max_context_tokens = 16385  consumed_context_tokens = 0  # Iterate over each key in the json file, which is the title of the paper  for paper,abstract in papers.items():  if(abstract != ""):  if paper in output:  print(f"Skipping {paper}")  continue  completion = client.chat.completions.create(  model="gpt-3.5-turbo-0125", # OpenAI model to use  messages=[  {"role": "system", "content": "You will have the role of a researcher that performs content analysis tasks."},  {"role": "user", "content":  "I need you to code a series of variables based on the abstract of a scientific paper. Next, I will describe you the variables. \n\n"+    "Variable \"research_approach\" which indicates whether the research is quantitative, qualitative or mixed. "+  "If the study has just one research method, it should be classified as Quantitative or Qualitative. "+  "If the study has more than one research method, it should be classified as Mixed.\n\n"+  "Variable \"research_method\" which indicates the type of the main research method used to collect data. "+  "Answer with one of the following values: "+  "Methodological advances, Systematic literature review, Meta-analysis, Big data, Experimental, Ethnography, Observational analysis, Case study, "+  "Discourse and textual analysis, Focus group, Interview, Survey, Content analysis, Network analysis. " +  "The values are ordered by descending priority, in case more than one research method is detected.\n\n"+  "Variable \"research_method_explanation\" where where you return an array with the text fragments used to determine the value of \"research_method\". "+  "Include up to 10 words per text fragment.\n\n"+  "All the variables have been defined. This is the content to analyze (between triple quotes): \n"+  f"\"\"\"{abstract} \"\"\"\n\n"+  "Return the response in JSON format. Do not include the content to analyze in the response." }  ],    response_format={ "type": "json_object" },  temperature=0  )  print(abstract)  response = completion.choices[0].message.content  print(response)  # Parse the JSON response as a dictionary  response = json.loads(response)  output[paper] = response  # Save the output as a json file  with open(output_file, 'w') as f:  json.dump(output, f, indent=4)  consumed_context_tokens += completion.usage.total_tokens  print(f"Total tokens consumed: {consumed_context_tokens}") |

Note: This source code implements the protocol for coding abstracts using two variables. Input abstracts and output file are in JSON files defined in the initial sentences of the program.
